# Shared intentionality, reason-giving and the evolution of human culture

**DOI:** 10.1098/rstb.2020.0320

**Published:** 2022-01-31

**Authors:** Cathal O'Madagain, Michael Tomasello

**Affiliations:** ^1^ School of Collective Intelligence, Université Mohammed VI Polytechnique, Ben Guerir, Morocco; ^2^ Department of Psychology, Duke University, Durham, NC, USA; ^3^ Developmental and Comparative Psychology, Max-Planck-Institute for Evolutionary Anthropology, Leipzig, Germany

**Keywords:** common knowledge, shared intentionality, cooperation, cumulative culture

## Abstract

The biological approach to culture focuses almost exclusively on processes of social learning, to the neglect of processes of cultural coordination including joint action and shared intentionality. In this paper, we argue that the distinctive features of human culture derive from humans' unique skills and motivations for coordinating with one another around different types of action and information. As different levels of these skills of ‘shared intentionality’ emerged over the last several hundred thousand years, human culture became characterized first by such things as collaborative activities and pedagogy based on cooperative communication, and then by such things as collaborative innovations and normatively structured pedagogy. As a kind of capstone of this trajectory, humans began to coordinate not just on joint actions and shared beliefs, but on the reasons for what we believe or how we act. Coordinating on reasons powered the kinds of extremely rapid innovation and stable cumulative cultural evolution especially characteristic of the human species in the last several tens of thousands of years.

This article is part of a discussion meeting issue ‘The emergence of collective knowledge and cumulative culture in animals, humans and machines’.

## 1. Introduction

It is widely recognized that human capacities for coordination and collaboration, as we find in joint action and shared intentionality, are some of our most distinctive psychological traits. But the role these capacities play in the generation of human culture is underexplored. Tomasello *et al*. [1] proposed that human cultural transmission was distinguished from that of other species by the accumulation of modifications over time via the so-called ‘ratchet effect’, leading to what is known as ‘cumulative culture’. One can see such a process in the historical development of all kinds of human tools and technology, as well as social structures and institutions. In cumulative culture, each generation builds on the innovations of the previous one, so that superior modifications are preserved and inferior ones left behind, hence ‘ratcheting up’ the skill or technology that is passed along. What is inherited is the product of several generations of innovation, and often goes beyond what any generation could have produced on their own. The cultural ratchet can be seen as a kind of collective pooling of knowledge and cognitive resources in the social group, as individual innovations are adopted and built on by everyone else, such that everyone benefits from one another's creativity.

In what follows, we propose an evidence-based account^1^ of how human cumulative culture evolved from the emergence of joint and shared intentionality, with a focus on the relatively recent role of coordination around reasons in this trajectory. In the 6 million years since humans have been on their own evolutionary pathway, human cultural transmission appeared and evolved (see [2,3] for two accounts of that process), and humans' unique social-cognitive skills of shared intentionality developed in parallel, in ways that have enabled that evolution. Arguably, the earliest hominins began with an ape-like form of culture based on individual innovation and learner-based forms of social learning. As cooperative communication and shared intentionality evolved in these groups, it enabled teacher-led forms of pedagogy, and then cultural processes characterized by collaborative innovations and normatively structured pedagogy. Finally, as a kind of capstone of this trajectory, there emerged a process that has not previously been discussed in this context: the human ability to coordinate around common reasons for their actions. The result was that actions were selected not just because of their apparent success or frequency, but because they came with convincing reasons. It is these ‘reason-based’ forms of transmission, and the ability to coordinate on joint reasons for action, that underlie the kinds of extremely rapid innovation and stable cumulative cultural evolution characteristic of the human species in the last dozen or so millennia.

## 2. Early hominins’ chimpanzee-like culture

Early hominins probably began with ape-like forms of culture and cultural transmission—which already involve simple forms of social learning. Chimpanzees live in social groups that display a variety of behavioural traditions, such as termite fishing and nut-cracking [4], which seem to be maintained by processes of social learning [5]. There is some controversy over the precise nature of this social learning. One possibility is ‘emulation learning’ in which the learner aims at reproducing the same outcome as another individual, but does not copy the actual technique used by the other to achieve that outcome. Another is ‘imitative learning’ in which the learner achieves the same outcome as another by copying the specific behaviour they use to achieve that outcome. While the skills learned in these contexts are often skills that could likely have been figured out by an individual chimpanzee on her own given enough time (hence calling into question the importance of the social learning component; [6]), on the whole, there is little doubt that chimpanzee individuals do in fact learn many of their skills from their peers.

Nevertheless, the kind of social learning available to chimpanzees can be clearly distinguished from that of humans. Experimentally demonstrated phenomena of chimpanzee social learning are especially relevant in this context. Haun *et al*. [7] found that chimpanzees are influenced in their social learning by the *number* of different demonstrators they observe performing some activity: not just how many times the individual sees the activity being done in total, but rather by how many different individuals they see doing it (the so-called majority bias, also seen in humans). However, chimpanzees are reluctant social learners when they have already learned to do something successfully on their own. In another experiment, Haun *et al*. [8] found that when chimpanzees had already learned to solve a problem one way and they then saw three other individuals solve it a different way, they stuck with what they knew worked individually, even if the new solution would lead to a greater reward (while human children switched) (see also [9,10]). Chimpanzees will therefore learn from others, but only if they do not already have a solution to a problem. Chimpanzees are of course prolific individual innovators. There are many relevant observations from the wild (e.g. [11]), and the evidence is especially clear in captive chimpanzees who invent creative solutions to experimental problems with regularity (e.g. using water as a tool; [12]). However, this inventiveness is also prototypically individual—there is no reliable evidence that chimpanzees collaborate to innovate, especially if we use as a criterion that individuals create a problem solution together that neither could create on its own. The basic psychological processes underlying chimpanzee culture therefore seem to be (i) individual innovation and (ii) conservative social learning, which is to say learning from others only when there is no existing solution.

There is no reliable evidence that these basic processes can lead to human-like cumulative cultural evolution [13], and indeed it is understandable why not. If individuals adopt a new technique only if they do not already have one, then this pattern will quickly lead to a deadlock of innovation. As soon as one individual in a group solves a problem, everyone in the group will copy the solution. Now, everyone has a way to solve the problem. Even if an outsider or lone innovator produces a novel technique that is more effective, others will not copy it since they already have a solution at their disposal.

Let us suppose that the last common ancestor of humans and other great apes (i.e. chimpanzees and bonobos) some 6 million years ago lived in social groups characterized by these kinds of cultural processes. This description almost certainly held for the first several million years of the hominin line as manifest in Australopithecines. Virtually no theorist posits anything different, as there is no artefactual evidence to suggest anything special in their cultural organization. Between the late Australopithecines and early *Homo*, around 3 to 3.5 million years ago in Africa, there emerged some new toolmaking behaviour [14]. Stone tools began being produced. Experimental studies of the ‘Oldowan’ tools produced by *Homo* by contemporary humans demonstrate that they are not particularly cognitively demanding (flakes are made in a fairly haphazard striking together of stones; [15]), and indeed with minimal experience, a human-raised bonobo has made Oldowan-type tools [16]. It is thus possible that Oldowan tools were mainly made by early individuals on their own, using only outcome-based processes of social learning, such as emulation learning. Oldowan tools were followed by early Acheulian tools, which were used for some time by various species of *Homo*, including *Homo erectus* as they spread out over the Old World. These tools are more challenging to make, but there was little innovation in them over hundreds of thousands of years. It is reasonable to suppose thus that both Oldowan and early Acheulian tools represent a continuation of the chimpanzee-like pattern of individual innovation plus conservative social learning [17].

## 3. Demonstrative pedagogy: coordinating on actions in the middle Pleistocene

The earliest evidence of cumulative culture appears in the middle Pleistocene of Africa and Europe (roughly 750 000 to 125 000 years ago, before the flourishing of modern humans) with the ushering in of late Acheulian technology, involving stone tools that are particularly difficult to make. Experimental studies with contemporary humans have investigated what it would take to become proficient in the use of different sorts of tools, and they indicate that many would be difficult or impossible to learn either individually or through simple observation [18,19]. In addition to these complex purely stone tools, late Acheulian technology includes composite tools that require preparation of different component parts before they are put together in some new way, such as the hafting of stones onto sticks in making such things as stone-tipped spears or hammers [20]. These are also extremely difficult to learn through observation only (especially the hafting process itself which requires the skilful use of some kind of adhesive), indicating a technology that has been developed over several generations, and one that requires something more than simple observation to master.

In their original paper arguing for cumulative culture via the ratchet effect in humans but not other apes, Tomasello *et al.* [1] pointed to the literature on social learning existing at that time and concluded that humans' more technique-based (as opposed to outcome-based) forms of cultural learning might explain this kind of accumulation of innovation. But research since that time has shown much greater skills in non-human animals than previously supposed—such as chimpanzees learning nut-cracking techniques from one another (for a review see [21]). Since there is no evidence for cumulative culture in the same group, one conclusion is that by themselves processes of social learning, including imitative learning, are not sufficient to produce a robust ratchet effect and cumulative cultural evolution. What is needed, we suggest, is a distinctive kind of collaborative helping, that depends on human capacities for coordination. This is that experts are incentivized to seek out novices and make sure that they learn to become proficient themselves—deliberately teaching them. The kind of innovations we see in late Acheulian technologies described above cannot be acquired by passive observation. They require a teacher to monitor a learner's progress, recognize when a novice needs help, and to demonstrate difficult techniques in a way that is sensitive to whether the learner is keeping up. For example, the teacher may slow down for the difficult parts, and attend to whether the novice is attending.

Late Acheulian technologies also change much more rapidly than previous technologies, with good evidence for the accumulation of modifications in particular technologies over time to meet novel exigencies. The emergence of intentional teaching can explain this. If we have the urge to deliberately teach others when we believe ourselves to have superior expertise (perhaps because we need proficient collaborators to help us with complex tasks) then the stage is set for others to adopt new solutions even if they already have a solution of their own. The deadlock described above—where once everyone in a group has their own way to solve a task, new solutions will not appear—can be broken by this kind of deliberate teaching. Even if I have my own solution, if you are eager to get me to adopt your one, this puts pressure on me to switch methods. The result is that individual innovations can now be spread in a group even when the group already has a solution at their disposal—and solutions can improve over time. Intentional teaching therefore offers an explanation for two features present in Acheulian technologies—the difficulty with which they are acquired by passive observation, and the accumulation of improvements in those techniques over generations.

This kind of helping—intentional teaching—depends on various elements of collaborative communication that emerge in early childhood. In prototypical human teaching, experts know they know more than others, and they seek out novices (even if only their children) and make sure that they learn to become proficient themselves [22]. This demands relatively sophisticated ‘mind-reading’, insofar as the teacher needs to evaluate their own knowledge-state and that of the novice, to be able to tell that the novice knows less than they do. It also requires ‘cooperative communication’, such as informative pointing and pantomiming gestures used to helpfully demonstrate a skill. And it requires that both participants are coordinated around an immediately observable joint task with mutual knowledge of their shared goal, so-called ‘personal common ground’ [23]. All of these abilities emerge in human ontogeny early on—pointing and joint attention by the end of the first year [24] and iconic gestures by the second year [25]. Equally, very young children will themselves inform others helpfully and selectively depending on the others' knowledge [26], even preferring to teach skills that they themselves found difficult to learn, demonstrating a sensitivity to when their help is needed [27].

It is intuitive to suppose that the origin of these abilities is in the kind of coordinated action that allows us to accomplish tasks that neither could accomplish alone. Tomasello [28] proposed that this kind of coordinated action first emerges in a context of ‘obligate collaborative foraging’, in which the resources required for survival can be only secured through collaboration. If the only prey around are large enough that we cannot capture them on our own, then collaboration becomes a necessity. If we collaborate on a hunt, we can capture a larger prey than either of us could capture on our own; if we collaborate on moving a log, we can move a heavier log than either can move alone. A context of obligate collaborative foraging therefore creates pressure for the selection of abilities to coordinate action for collaboration, and the downsides of collaboration (such as the risk of free-riding) are offset by the reward of surviving in such an environment. Once the socio-cognitive abilities required for collaboration are in place, skills that require collaborators (such as joint hunting) will develop. And this creates an incentive to teach others—if I need a good hunting partner, I have an incentive to teach a novice the skills that I have acquired, so that s/he can help me in the joint tasks I have to undertake. A context of obligate collaborative foraging can therefore bring about both the socio-cognitive resources necessary for intentional teaching, and also create an incentive for experts to teach novices, so that they will have capable collaborators.^2^

Clear evidence in the fossil record for collaborative foraging begins in the middle Pleistocene, with especially clear evidence for *Homo heidelbergensis* at around 400 000 years ago [31]. Tomasello [32,33] speculates on this basis that it was during the middle Pleistocene that hominins became proficient with the cognitive skills required for collaborative foraging: joint attention, joint goals and joint decision-making, and the cooperative communicative skills described above. And so we think that the first signs of the cultural ratchet appear due to the emergence of the kind of cooperative communication that arises given environmental pressure to collaborate, and the intentional teaching this environment enables and selects for.

## 4. Normative pedagogy: coordinating on rules in modern humans

Anatomically modern humans emerged sometime around 200 000 years ago, and by the upper Pleistocene (125 000 years ago), they were living in distinct cultural groups. Each group had its own ways of doing things, including its own distinctive tool kit with tools for all kinds of functions, many of which were opaque without some form of observation or instruction. These various tools underwent relatively rapid modifications over time. With modern humans, the cultural ratchet is in full force. And while simple collaborative action was already a feature of earlier human society, it was during this period, we suggest, that the emergence of linguistic abilities allowed collaboration to extend to the level of plans and ideas—what could be called the emergence of ‘collaborative thinking’.

A distinctive feature of these modern cultural groups is the social structures they created to coordinate their intragroup interactions: cultural conventions and norms that governed all aspects of life in a modern human cultural group. These conventions and norms are collectively created and maintained, motivating individuals to follow and enforce norms governing issues such as mating and the sharing of food. They are not simply patterns of behaviour that are imitated and spread in the group, which we find in many non-human species [34]. Rather, they are behaviours that are governed by rules, often linguistically expressed, which are enforced by the group and deviance from which is chided by others.

The transition to a society that is governed by shared norms or rules can be thought of as a transition from joint intentionality to collective intentionality [32,33]. Collective intentionality involves group members expecting one another to conform to the ways of the group—the practices and beliefs that form the ‘cultural common ground’ [23]. In the study by Haun *et al*. [8] cited above, in which chimpanzees were inclined to stick to what already worked, children abandoned their previously successful behaviour to adopt that of three other children who did it equally successfully but a different way. They did this even more emphatically if the other children watched, suggesting active conformity to forestall criticisms (see also [35]). This tendency can also be seen in contemporary children's so-called ‘overimitation’, in which they imitate irrelevant parts or aspects of a demonstrated action—which apes do not do [36]—and they even claim that this is how things *must* be done [37].

Modern human cultural life also comprised a transition from gestural forms of communication to linguistic communication. Linguistic communication operates with basically the same cooperative motives as gestural communication, but it has distinctive advantages. One is that it can be used with in-group strangers much more effectively than can gestural communication, which relies to a great degree on individuals having shared experiences with one another. Another is that linguistic communication allows for the expression of generalized ‘rules' that apply not only to the current situation but to a broad range of situations—including situations absent from the context of conversation. This is evident in the way that young children take instructions taught verbally to be ‘generalizable’ [38]. In linguistic pedagogy, the teacher's instructions are understood not only to be about the concrete situation at hand (e.g. ‘Strike this stone here now’), but rather as instructions on the culture's knowledge of how things work in general (e.g. ‘To make these kinds of tools, one must strike these kinds of stone like this.’). Studies by Butler and colleagues explicitly contrast a situation in which children observe an adult doing something—in which case they might learn it but not generalize it to similar artefacts—with a situation in which the adult instructs them, in which case they not only learn but also generalize [39,40]. To our knowledge, great apes neither teach nor learn in this culturally generalizable way. By contrast, contemporary children do learn in this way from around their third birthdays.

Normative or rule-based pedagogy will clearly increase the efficiency of cultural transmission. Suppose you are demonstratively showing a novice how to gut a fish, using the efficient technique of making a small hole in the neck and squeezing the guts out through this hole. If your student fails to take the demonstration to be a generalization, they may fail to realize that it applies to other fish—which would really mean failing to learn the rule (they might think it applies only to *this fish*, [41], §28). But even if the student takes the instruction as a generalization, what if the technique only applies to some particular species of fish? Or some particular genus of fish? Teaching where the rule applies and where it does not will take endless work if all that is available is case by case demonstration. With generalizations over category terms in a shared language this is vastly simplified, since we can say things like ‘all flounder can be prepared for preservation using this method’, or ‘all flatfish can be prepared this way’. The great utility of a shared set of category terms for the kinds and species in your environment, and the ability to make generalizations over those categories, is that it maximizes the efficiency with which reliable and informative rules can be learned and communicated [42,43].

Once we have rules articulated in a common language, we can also collaborate on improving the rules themselves, including joint planning. This is a form of coordination in which it is not concrete objects like spears or mammoths that we are coordinating around, but abstract objects such as rules, expressed in language, that we jointly act upon. When children are around 4 years of age and begin referring recursively to their own assertions and proposals (using discourse demonstratives like ‘that's a good idea!’), entities like rules and plans themselves become the focal point of a conversation, and the object of joint action. This may be called ‘joint attention to mental contents' [44], since it results in the ‘contents' of our minds, like our internal beliefs and plans, to become the focal point of joint attention and action. While one person might articulate the belief or rule ‘to make a spear, you must use hardwood’, another might object ‘*that's* not always true, you can use a softwood if the spear is big enough’. Now it is not the actual physical spear that is the object of joint focus, but the rule that is believed to govern its creation.

Once the rules or norms of a community are available for joint exploration in this way, members can begin to collaborate not just on practical tasks (like creating an arrow together), but on the rules that are taken to govern those tasks. This means that the rules themselves become subject to the cultural ratchet, improving as they are passed across generations.

Notably, at around the same age that contemporary human children are showing tendencies to conform normatively (i.e. as they approach school-age), their ability to create things collaboratively becomes much more pronounced. They begin to create and enforce social norms or rules to regulate their peer play (e.g. rules of a made-up game that they insist novices *must* follow; [45]). And their ability to co-construct novel solutions to problems that neither individual could construct on her own also becomes more pronounced [46]. The ability to coordinate attention and action on rules, jointly commenting on and constructing rules and plans for action, we suggest, forms an essential element in this transition. Once a plan of action can be articulated and jointly developed, coordinated action can be executed more efficiently. It can include contingency plans to be executed even if the parties to the plan have to act separately (you go to the mountain and I'll go to the meadow, and whoever finds the deer tracks first make a blast on the horn and the other person will come to that location). Without the ability to articulate plans in advance, collaboration cannot extend beyond joint action on a concrete task that both agents are attending to. But when the rules and plans can be articulated and commented on, they can be refined in light of both parties knowledge and insights, and also enable actions that can be executed without both parties being continually in contact.

On this view, modern humans in the upper Paleolithic began to innovate together not only on practical tasks like collaborative foraging, but also by collaborating on the creation of new procedures and rules for the practices they inherited, and jointly refining those rules as they are passed across generations. This further reinforces the cultural ratchet, leading to new sources of innovation and an inevitably faster pace of cumulative cultural evolution.

## 5. Epistemic pedagogy: coordinating on reasons in early civilization

Then, sometime after 50 000 years ago, in some localities, there was an increase in the pace of innovation and cultural accumulation in many human populations. Part of the explanation for this intensification, we propose, lay in the emergence of a more complicated kind of coordination again. Now not only rules and plans can be proposed and commented on, jointly constructed through conversation, but also the *reasons* for why we might adopt one or another plan or rule become subject to joint attention and action. All great apes are capable of making causal inferences [47], but with the advent of linguistic communication of a particular degree of sophistication, humans became able to communicate their causal reasoning to others. Tomasello [32] argued that such reason-giving behaviour must have gotten off the ground in the context of collaborative decision-making in which participants agreed not to try to dominate the interaction individually but rather to respect and abide by those ideas and proposals backed by the best reasons, whoever proposed them—as that would lead to the highest probability of collaborative success. But reason-giving will also have had a major impact on cumulative culture. It allows for the transmission not only of rules, beliefs and techniques, but for the *reason why* particular rules or techniques are adopted. Recalling Plato's view that knowledge depends on understanding the *reasons* for our claims and proposals [48], we could call this ‘epistemic pedagogy’.

### (a) Selection by reasons

We saw above how, once individuals become enthusiastic teachers of their own solutions (for the sake of creating good collaborators), the deadlock that can arise from conservative social learning can be broken. But this will create a possible excess of teachers: once the technologies we have developed require good collaborators to be effectively built or used, individuals will want students to join them to learn their techniques. If potential students recognize the skill that I have is valuable, they will want to learn from me—and this creates a collaborator for me, as we work together on tasks that I would otherwise have to do alone. Teachers therefore have an incentive to be recognized as providing good instruction and skills, and learners must choose from whom to learn.

The reasons a teacher offers as a justification for their technique can provide a student with a way to decide whose technique to try to master. Suppose that we are looking for a good way to tie up a canoe. We see that our peers have tied up their canoes in several different ways, including a loose loop, or tying it in several difficult to unravel knots. One of our peers—a minority, and perhaps one who has not yet distinguished themselves in any way in the group in terms of individual success—has looped the rope just once but used a single special knot. To find out why, we ask the person who made this knot. She tells us ‘well, this knot won't be undone by the pulling of the tide on the canoe, but it can be quickly removed by tugging on this loop—allowing me to undo the canoe easily when I need it’. If we ask other canoe-owners why they have tied their own canoe as they have, and their explanations are less convincing (e.g. ‘it's easy’, or ‘I couldn't think of any other way to do it’) the provision of a good explanation has now itself become a selection mechanism for a particular technique to be transmitted (for relevant new work on what counts as a good explanation see [49]). Our remarkable ability to distinguish good from bad reasons is sometimes called ‘epistemic vigilance’ and has been argued to function primarily to allow us to detect poor claims [50]. But beyond this use, it will also allow us to identify the best proposal when faced with many. Faced with a sea of enthusiastic teachers trying to convince us to adopt their innovations, an evaluation of their reasons will allow us to cut through the chaff.

Of course, recognizing a demonstrably more successful technique among the range of options could have the same effect [51]. However, demonstrating the success of a new technique before it can be adopted is not always going to be a practical option. What could demonstrate the effectiveness of tying up a canoe, except a storm in which any canoes that are not properly secured would be swept away and lost? Such a demonstration is something many communities will not be able to afford. If the stakes are too high (if failure means losing a canoe), we cannot afford to test and retest new techniques until we find the demonstrably best solution. Identifying the best solution on the basis of its rationale, on the other hand, is free.

### (b) Transmission by reasons

Epistemic pedagogy also leads to much more *stable* cultural transmission, including in new contexts. It is a common observation that when we mimic someone doing something without understanding its rationale, or the causal basis for the action, we might succeed and continue to succeed as long as the situation remains the same. Henrich [52], looking at the transmission of the preparation of manioc plants over generations where individuals clearly do not understand the causal reasons for various steps in its preparation, argues that effective cultural transmission does not require understanding why something functions as it does. More recently Derex *et al.* [53] have shown that cumulative innovation can take place in the absence of causal understanding. But what if the situation *changes* such that the individual needs to make an appropriate adjustment, but does not understand why she was using the technique that she had learned from her teacher? Perhaps the original materials are no longer available, or perhaps the environment has changed so that old tools need to be adapted to new tasks. To adapt a technique to new contexts, we suggest, it will often be necessary to understand why the technique she was using worked, so that causal knowledge can be used to properly adapt the technology. To return to Plato's original example, a traveller is at an advantage if he not only knows which road to take to get to Larissa, but also the reason why. Perhaps the reason is because Larissa is to the north and this is the road north. If the traveller sets out on the road but after some time gets disoriented and loses the trail, he will be able to reorient himself by heading north even if he cannot find the path. But if he does not understand the reason why this was the correct route to take in the first place, he will not be able to correct himself if he goes wrong and loses the trail.

The same principle applies to the transmission of technology. Imagine some modern humans several tens of thousands of years ago making and using hafted tools such as arrows with stone tips. The children as apprentices watch as the adults wrap the shaft beneath the arrowhead with sinew. Although it appears to be designed to keep the arrowhead on tight, its purpose is primarily to prevent the arrow shaft from splitting when the arrow hits its target. Now suppose the apprentice is displaced from the instruction of the adults and in a new environment is forced to use new materials to construct the arrow. If she thinks the reason the sinew was attached was to secure the head, she may use something like a copper ring to do the job, which secures the arrowhead but does not reinforce the shaft. And then on the first impact, the shaft will split. When the instruction comes with the reason, on the other hand, if the apprentice is forced to find a substitute for the sinew, she will find one that secures the shaft, rather than the tip—such as a cloth wrap. More generally, then, when a technology is transmitted, the community that can transmit the most causally accurate reasons along with the demonstration will be at the greatest advantage, and the technology can be adapted to new environments with new materials in such a way that is most likely to preserve the functions of different components.

We obviously have no way of knowing when such reason-giving behaviour came to play an important role in human cultural innovation and transmission or at which localities. One candidate would be the cultural explosion in Europe some 40 000 or 50 000 years ago. This cultural explosion obviously has many causes, but structuring collaborative and pedagogical interactions with language, especially reason-giving language, could potentially have played a role. A more recent likely locus would be in Mesopotamia and Egypt at the dawn of Western civilization some 10 000 years ago. We know that one of the drivers of the incredibly rapid cultural ratcheting during this time period was the exchange of ideas among different civilizations. These innovative ideas were typically communicated between civilizations not through extensive contacts among the group members, but rather by individual traders (often pastoralist herders). Because the materials available and the problem situations would have been different in the different localities, it would be especially important that the individual bringing the innovation should understand the reasons why original materials were used, in order to find appropriately similar substitutes in a new situation. Obviously, the contemporary forms of globalized cultural transmission are the ultimate result of this new way of doing things.

### (c) Ratcheting reasoning

Once not only tools and techniques, but also the reasons we have for adopting them are being passed along, the reasons too will be subject to the cultural ratchet. Each generation that receives a technique along with its explanation can improve and build on not just the technique, but also the explanation. Such cooperative thinking, as we may call it, both depends on common ground (what has been passed down to us from the previous generation) and contributes to it (and sends forward to the next generation). Suppose one generation believe that the arrows with hawk feathers fly best because hawks fly fast. An individual in the next generation realizes that the reason hawk feathers work well is because they tend to create a helical shape when attached to an arrow, and that if a turkey's feathers are twisted to create a helical shape they will work just as well, in fact better because they are more malleable. Once reasons are part of our common knowledge, this reason can be added to the shared pool of knowledge, augmenting everyone's ability to make good arrows given the greater abundance of turkey feathers over hawk feathers. Now that we all know, and we all know that we know, why some feathers work better than others in the flight of arrows, further individual or cooperative reasoning about, for example, the specific placement of feathers for maximal performance can take place.

At the limit, this process results not just in pieces of reasoning about the way the world works being inherited and refined, but our skills of reasoning themselves being improved via the cultural ratchet. When we are engaging in scientific reasoning today, we employ statistical tools of reasoning that are complicated enough that arguably no individual in the history of our species could have invented them in a single lifetime. Cooperative reasoning can provide a means for the selection and improvement not only of practical techniques, but ultimately for the selection and improvement of strategies of reasoning and the epistemic norms that govern their use in practice [54]. We teach our offspring to use techniques and tools of reasoning that have been refined by indefinitely many individuals over millennia of cultural transmission, and we have the ability to optimize the sequence in which they acquire these tools, dramatically augmenting our cognitive abilities over generations.

## 6. Conclusion

**Figure 1.**
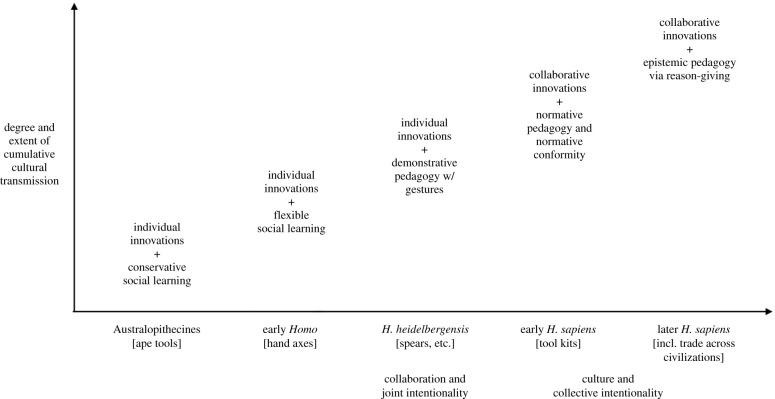
Overall schematic depiction of changes in the processes of coordination leading to different kinds of transmission in hominin culture over the last 6 million years.

Our overall account is summarized in figure 1. What we want to emphasize is that the cumulative cultural transmission of the human species has changed over evolutionary time, and these changes are based on changes in processes of innovation and transmission. Obviously, we cannot observe these processes in the artefactual record, and so we must use indirect methods. Most common is analysis of the nature of particular artefacts and what it takes to make them, perhaps combined with experiments with contemporary humans in making and using them (e.g. [18]). We have added here, in addition, behavioural experiments with great apes and children of different ages to add further indirect evidence to our inferences about human cultural processes.

We have mostly ignored here the social dimension of the process in the sense that the nature and pace of cultural transmission will be dependent on how often and in what ways individuals come into contact with one another. Thus, Hill *et al*. [55] have argued and provided evidence that familial and societal structures must be of a certain nature (e.g. population must be sufficiently dense, familial ties must be of a certain type, etc.) for certain types of cultural transmission to be viable. This would help to explain why we do not see a perfectly uniform ratchet across all of the various human populations that exist at a particular evolutionary period. In the end, we must integrate these kinds of social dynamics into our explanations of the collective knowledge and cumulative cultural evolution characteristic of the human species.

Theoretically, we believe that the framework of shared intentionality provides a useful one for characterizing the different ways that humans put their heads together—either simultaneously in collaborative inventiveness or across time in cultural accumulation—to do things that no one of them could do alone. It provides us with two different steps—joint intentionality among collaborating individuals and collective intentionality among the members of a cultural group—that helps, we would argue, to account for different forms of human culture across time. In any case, our most basic argument is that the nature of human cultural organization and collective knowledge, as well as cumulative cultural evolution, have changed significantly over the last 6 million years of hominins on planet Earth, and so it would behoove us to take account of this historical variation in our explanations. In addition, we have argued that a previously undescribed process—epistemic pedagogy using reasons—could explain some of the especially rapid cultural accumulation that seems to characterize some groups of humans in some locations in the last several tens of thousands of years.

## Footnotes

1 We draw primarily on evidence from behavioural experiments with great apes and human children, mostly to make claims about what kinds of collaboration, communication and social learning go together and how they do so. We also draw on some evidence from the development of human artefacts, but only illustratively, since the artefactual record of tool use and toolmaking over this timeline is quite complex, depending on many factors including the region of the world at issue. It is often the case that the processes we explore would depend on demographic or environmental features that we do not have space to discuss: our goal is to highlight the essential role played by the psychological factors we consider, not to show that they are sufficient to result in cumulative culture taken outside of an appropriate environment.

2 A note on the coevolution of culture and teaching: teaching can initially be supported by processes of kin selection, as adults instruct their offspring who then fare better and pass on their genes. But this process can then be generalized to others, perhaps as adults see the need for more knowledgeable and successful collaborators. If I teach another a skill that I have mastered, they can collaborate with me on the execution of tasks requiring that skill. As tools become more complex, groups that engage in intentional teaching will have an advantage over those that do not, since they have a higher likelihood of passing on technologies that have become opaque to an observer (for experimental evidence of this [29,30]). In this way, teaching and culture coevolve. It is only once skills have emerged within a community that require teaching to be transmitted that teaching can be selected for. The emergence within culture of certain skills is therefore what creates the evolutionary niche required to select for the teaching abilities required to pass them on.

## Data accessibility

This article has no additional data.
